# InfersentPPI: Prediction of Protein-Protein Interaction Using Protein Sentence Embedding With Gene Ontology Information

**DOI:** 10.3389/fgene.2022.827540

**Published:** 2022-03-28

**Authors:** Meijing Li, Yingying Jiang, Keun Ho Ryu

**Affiliations:** ^1^ College of Information Engineering, Shanghai Maritime University, Shanghai, China; ^2^ Data Science Laboratory, Faculty of Information Technology, Ton Duc Thang University, Ho Chi Minh, Vietnam; ^3^ Biomedical Engineering Institute, Chiang Mai University, Chiang Mai, Thailand; ^4^ Department of Computer Science, College of Electrical and Computer Engineering, Chungbuk National University, Cheongju, Korea

**Keywords:** protein-protein interaction, gene ontology, text mining, sentence representations, infersent

## Abstract

Protein-protein interaction (PPI) prediction is meaningful work for deciphering cellular behaviors. Although many kinds of data and machine learning algorithms have been used in PPI prediction, the performance still needs to be improved. In this paper, we propose InferSentPPI, a sentence embedding based text mining method with gene ontology (GO) information for PPI prediction. First, we design a novel weighting GO term-based protein sentence representation method to generate protein sentences including multi-semantic information in the preprocessing. Gene ontology annotation (GOA) provides the reliability of relationships between proteins and GO terms for PPI prediction. Thus, GO term-based protein sentence can help to improve the prediction performance. Then we also propose an InferSent_PN algorithm based on the protein sentences and InferSent algorithm to extract relations between proteins. In the experiments, we evaluate the effectiveness of InferSentPPI with several benchmarking datasets. The result shows our proposed method has performed better than the state-of-the-art methods for a large PPI dataset.

## Introduction

Protein-protein interaction (PPI) plays a vital role in cellular systems of organisms (Zhao et al., 2020). Most biological processes within a cell are induced by a variety of interactions among the proteins, such as signal transduction, immune response, and cellular organization (Sun et al., 2017). PPI detection is very important for researchers to study the properties of cellular systems and improve the understanding of disease and provide a basis for the development of novel therapeutic approaches (Liu et al., 2020).

Due to the importance of PPI in the field of biology, a variety of computational methods based on various sources of biological information have been proposed for PPI prediction. Researchers have been predicting PPIs using a protein sequence (Hashemifar et al., 2018; Li et al., 2018; Yao et al., 2019) and PPI network information (Liu et al., 2020; Yang et al., 2020). For example, in DeepFE-PPI (Yao et al., 2019), a new residue representation method named Res2vec is designed for protein sequence representation, combining effective feature embedding function and powerful deep learning technology to infer PPI. Research results of previous works (Hashemifar et al., 2018; Li et al., 2018; Yao et al., 2019; Liu et al., 2020; Yang et al., 2020) show that protein sequence and PPI network information based PPI prediction model can achieve high predictive accuracy, but they have high time complexity because computation is complicated by protein vectorized representations based on protein sequence information (Liu et al., 2020).

Gene ontology (GO) information is applied to PPI prediction (Smaili et al., 2018; Duong et al., 2019; Zhong et al., 2019). GO (Consortium, 2004) is a standard ontology that describes biological entities and relationships between them. It is organized as a directed acyclic graph (DAG), named GO graph. In a GO graph, each node is a GO term, and each edge between the nodes is the relationship between the terms. Since these GO terms are used to annotate biomedical entities, a protein is represented by a set of GO terms. Therefore, the semantic similarity between GO terms can reflect the properties of relationship between proteins to some extent. GO based methods can make accurate predictions at a lower cost, and they analyze the relationship between two proteins by comparing the similarity between GO terms (Consortium, 2017). Previous methods (Resnik, 1995; Lin, 1998; Pekar and Staab, 2002; Wang et al., 2007) compute the semantic similarity between two GO terms according to the structure of a GO graph. According to the similarities between two terms in GO, the semantic similarity between two proteins is calculated by AVG (Xu et al., 2008), Max (Pesquita et al., 2009), best match average (BMA) (Li et al., 2010), and so on. The structure-based methods are roughly divided into two types: node-based or edge-based. Node-based methods such as Resnik (Resnik, 1995) and Lin (Lin, 1998) focus on the information content (IC) of the most informative common ancestor (MICA). Edge-based methods such as Pekar (Pekar and Staab, 2002) consider the longest path from the nearest common ancestor to root, the longest path between GO terms and their common ancestor. Wang and others (Wang et al., 2007) developed a hybrid method to calculate semantic similarity using the topology of GO graph structure, and they consider the different kinds of relationships in GO graph. However, GO structure-based methods mainly consider the locations of GO terms in the GO graph, they did not fully mine information of the GO graph and gene ontology annotation (GOA). GO graph includes the term-term relations of GO terms, while GOA includes the term-protein annotations between GO terms and proteins (Zhong et al., 2019). Each GOA record also contains evidence from published experiments or inferences using computational methods (Liu and Thomas, 2019). By fully mining the GO graph and GOA, relevant information can be captured from term-term relationships and term-protein annotations relationships to predict PPI. Therefore, in order to make reliable PPI predictions, we need to fully mine relevant information of the GO structure and GO annotation at the same time (Mazandu et al., 2017).

Text mining techniques have been applied to extract protein information and construct PPI networks (Ma et al., 2019). A text mining method can make full use of a great quantity of literature to reveal potential protein-related knowledge. Deep learning architecture can utilize multiple hierarchical layers to extract effective features (Jin et al., 2020). Recently, some researchers used word embedding techniques to represent proteins with word vectors based on a large scale of corporation and predicted PPIs based on the protein vectors (Smaili et al., 2018; Duong et al., 2019; Zhong et al., 2019). When they generate the protein vectors, the relations between GO terms (for short, named GO-GO relations) or relations between proteins and GO terms (for short, named protein-GO relations) were considered. But they did not fully utilize the protein-GO relations, GO-GO relations, and protein-protein interactions together to construct the PPI prediction model.

In this paper, we propose InferSentPPI, an efficient supervised sentence embedding based PPI prediction method by capturing information of GO structure and GO annotation. Comparing with the normal corpus-based approach, InferSentPPI considers three kinds of relationships together, which are protein-GO relations, GO-GO relations, and protein-protein interactions. To utilize protein-GO relations, InferSentPPI regards a protein as a sentence, and it represents protein with GO terms. Its related GO term’s vectors are the words that make up the sentence. To utilize semantic relations between GO terms, the GO term vectors are created by Word2vec from the GO graph structure. InferSentPPI uses the modified supervised sentence embedding model InferSent (Conneau et al., 2017), which can capture associations between GO terms annotating the proteins in the PPI datasets. Therefore, our method can fully mine the information of GO graph, GO annotation, and PPI information to obtain high quality protein vector representations for reliable PPI prediction.

The main contributions of this study are as follows:

(1) A new protein sentence embedding based PPI prediction method with GO information was designed and implemented.
(2) Three kinds of biological relationships are applied to PPI prediction together, which are protein-GO relations, GO-GO relations, and protein-protein interactions. It fully mines the information of GO graph, GO annotation, and PPI information to obtain high quality protein vector representations for improving the performance of PPI prediction.
(3) An efficient GOA preprocessing method, generation of weighted protein-GO annotation axioms for protein sentence representations based on the reliability, is proposed for improving the performance of PPI prediction.

## Methods

### Overview

**FIGURE 1:**
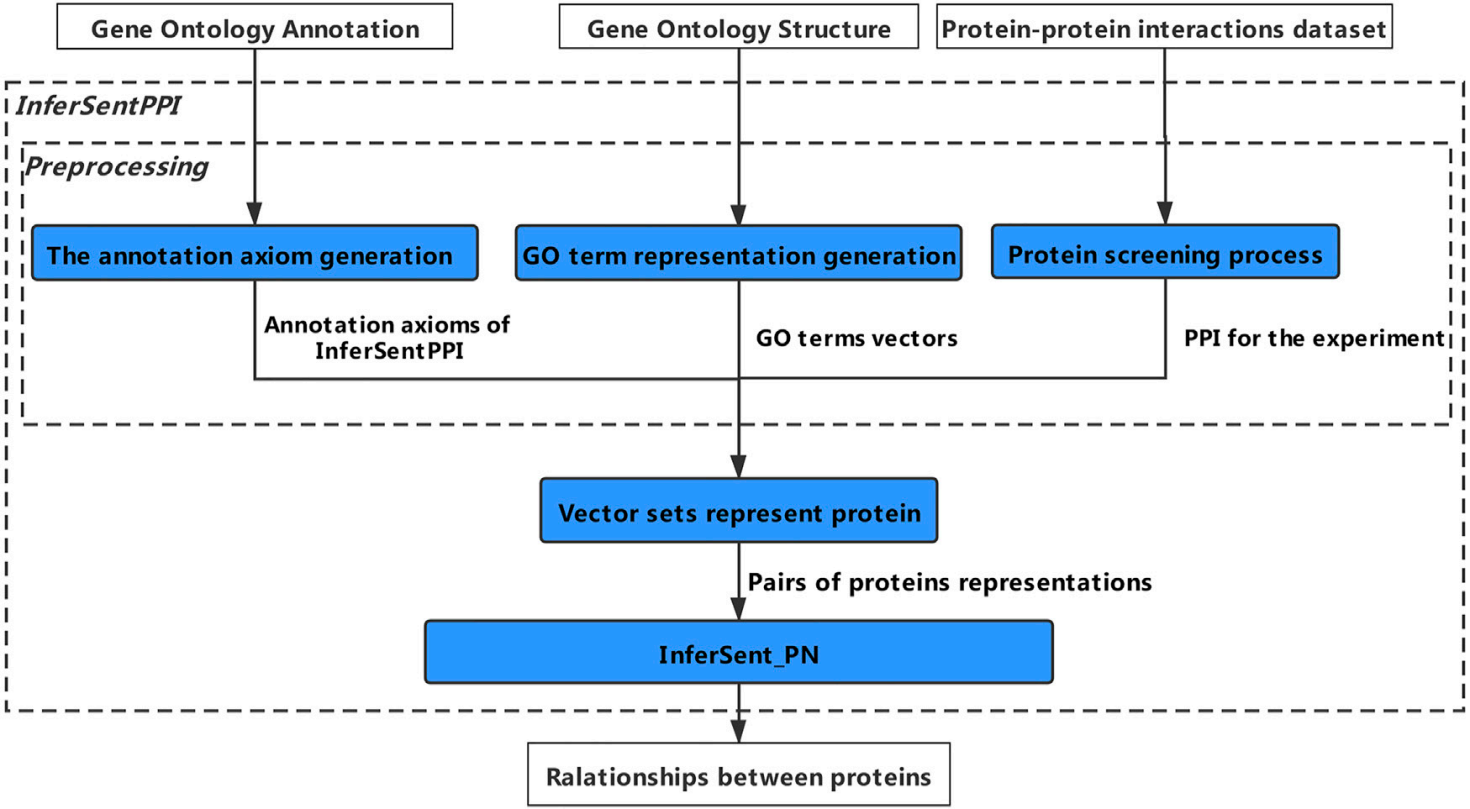
The workflow of InferSentPPI method.

InfersentPPI includes three main stages: preprocessing, protein sentence representation, and InferSent_PN as shown in Figure 1. In preprocessing, we exact the protein-GO annotation axioms and GO term vectors from GO graph and GOA separately, which are supposed by GO resource. We also extract PPIs from the PPI database. In protein sentence representations, protein is represented by its related GO term vectors first. Then protein sentence corpus is generated, which is composed of pairs of protein sentences and PPI labels. In the third stage, we apply InferSent_PN model to predict PPIs, which is constructed based on protein sentence embedding. Finally, we get relationships between proteins, PPI positive or PPI negative.

### Preprocessing

Preprocessing consists of three parts: annotation axiom generation, GO term vector generation, and protein screening process. Annotation axiom generation is a task to extract the relationship between protein and GO terms and represent protein with its related GO terms. GO term vector generation is a word embedding based task to mine semantic information between GO terms from GO structure. The protein screening process is a task to find the available PPIs from the PPI databases.

#### Annotation Axiom Generation

GOA includes the term-protein annotations between GO terms and proteins. Therefore, we extract the reliable annotation relationship between GO terms and proteins from GOA.

To obtain reliable protein-GO annotation axioms, we filter GOA records according to reliable evidence. The record of protein-GO annotation axioms from GOA is defined as the following: Protein_GO_record (GO, protein) = {GO ID, protein ID, Evidence Code}.

**FIGURE 2:**
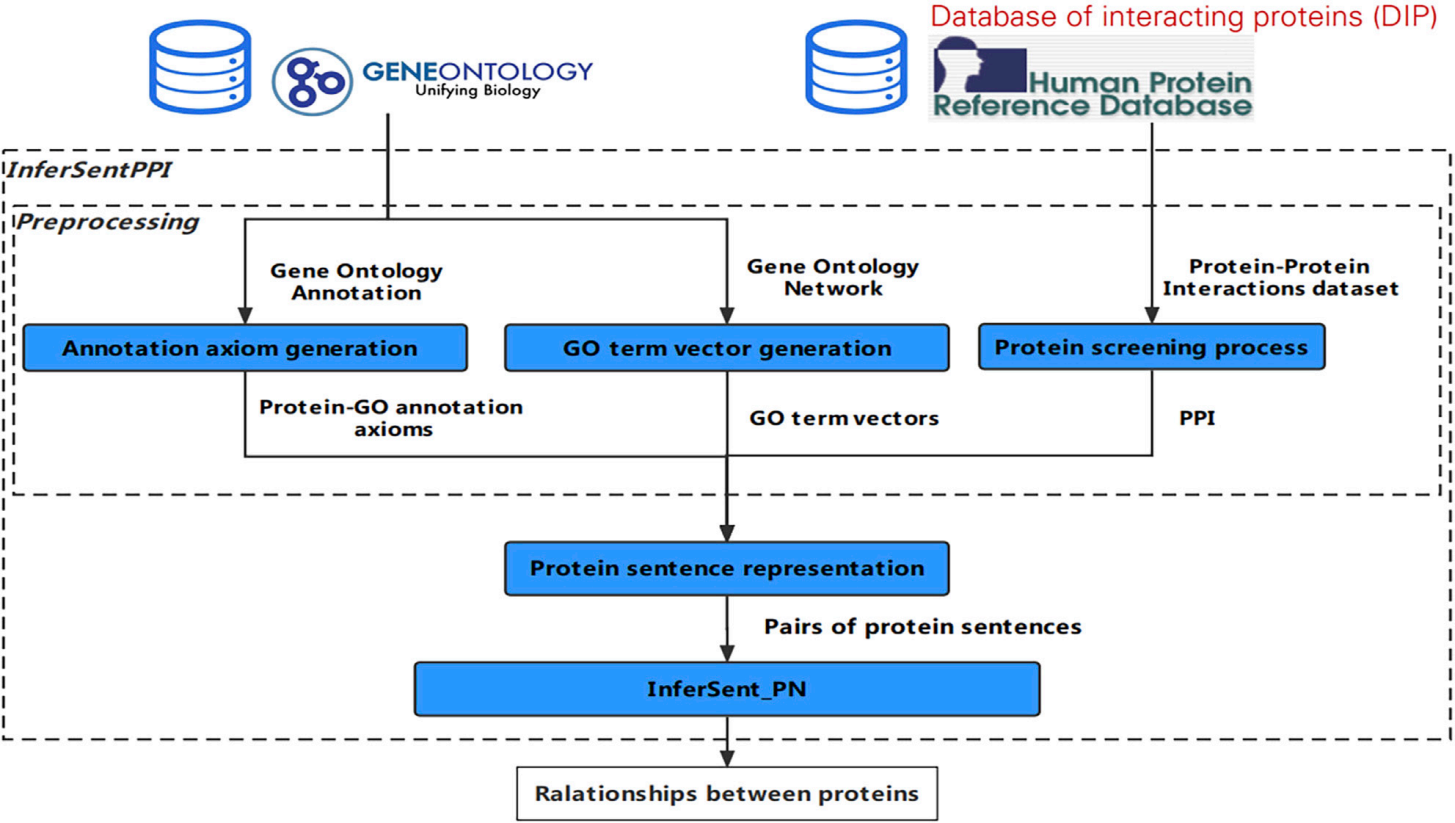
The workflow of the annotation axiom generation.

The specific generation steps are as shown in Figure 2. First, only the reliable protein-GO annotation axioms are needed; thus, we delete the annotation records without reliable evidence whose “Evidence Code” field value is “IEA” or “ND”, and obtain the reliable GOA record file. The evidence code “ND” indicates that biological data of the gene or gene product being annotated is not available. The evidence code “IEA” indicates the protein-GO relation is not manually reviewed and cannot generally be traced to an experimental source. Here, reliable protein-GO relations from an experiment directly supporting or it is manually reviewed. So, evidence code can reflect the reliability of protein-GO relations effectively. Second, based on the reliability, we give a weight to the protein-GO annotation axioms. We keep the protein-GO annotation axioms that appear many times in the GOA record file and note the repeated times as the weight. If an annotation record appears many times, it means that the correlation between them can be proved many times in different papers. Therefore, the number of repetitions can be used as a quantitative index to evaluate the reliable evidence of the annotation record. The final protein-GO annotation axioms with different weights are called “PGAA_ Weight”. We also generate protein-GO annotation axioms without the weight, named “PGAA_ noWeight”.

#### GO Term Vector Generation

GO graph includes the semantic relationships between GO terms. Thus, we apply the Word2vec (Mikolov et al., 2013) algorithm to generate the GO term vectors learning the network structural information from GO-GO relations. GO term vectors imply semantic relationships between GO terms because vectors are generated based on GO graph. Learned vectors can be applied to a variety of bioinformatics applications, such as predicting protein-protein interactions. This method is already used by other papers to generate the semantic GO term vectors and proved to be useful in predicting protein-protein interactions, such as Onto2vec (Smaili et al., 2018). GO term vector GOV can be specified in the following form:

$$\text{GOV}=\left({\text{v}}_{1}{\text{, v}}_{2}{\text{, v}}_{3}\text{,}......{\text{,v}}_{\text{n}}\right)$$

where v_1_, v_2_, v_3_, … ,v_n_ are the components of GOV.

#### Protein Screening Process

To obtain available PPI datasets for constructing the Infersent_PN model, first we select the PPIs whose protein can map UniProt ID because proteins without UniProt ID cannot find their related GO terms. Then we select the PPIs whose proteins have their related GO terms and can be represented by GO terms.

### Protein Sentence Representation

Protein is annotated by several GO terms. Therefore, protein can be represented by a set of vectors of a GO term. An n-dimensional protein vector P can be specified in the following form:

$$\text{P}=\left({\text{GOV}}_{1}{\text{, GOV}}_{2}{\text{, GOV}}_{3}\text{,}.......{\text{,GOV}}_{\text{n}}\right)$$

where GOV_1_, GOV_2_, GOV_3_, … ,GOV_n_ are the GO term vectors.

In this work, a protein is regarded as a sentence; a GO term is regarded as a word; a sentence corpus PC is composed of protein sentences and relationship label between protein pairs. PC can be specified in the following form: PC=(P_i_, P_j_, L) where P_i_ and P_j_ are any two proteins, and L is the relationship label.

**FIGURE 3:**
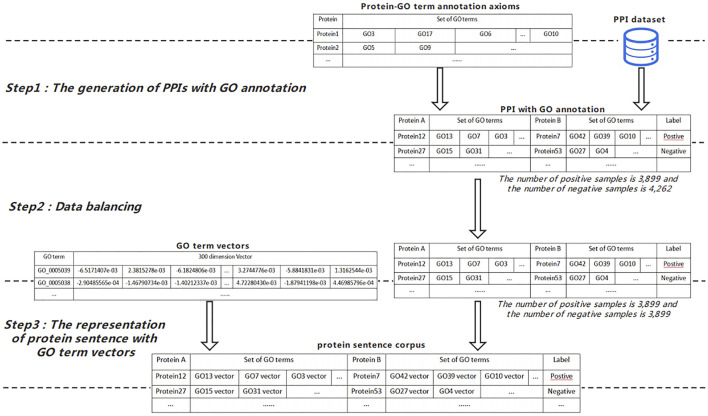
The workflow of the protein sentence representation.

To get the protein sentence corpuses used in the InferSent_PN model, the following three steps as shown in Figure 3 need to be completed: 1) Step 1, we combine the annotation axioms generated by preprocessing module with the PPI dataset for the experiment to get the PPI data with GO term notes. Obviously, we take PPI data with GO term notes as sentence corpus. 2) Step 2, we sample the same number of positive and negative protein interaction pairs from PPI data with GO term notes to be used in next step. 3) Step 3, we combine the representations of GO terms generated by the preprocessing module with PPI with GO term notes to obtain the training data of InferSent_PN, which is composed of pairs of protein sentence representations and relationship labels between protein pairs.

### InferSent_PN

To detect the relationship between PPIs, we proposed a new prediction method InferSent_PN, which is based on the InferSent algorithm (Conneau et al., 2017). InferSent (Conneau et al., 2017) is a classification model based on neural network structure for Natural Language Inference (NLI) tasks, and the first layer is the word vectors of all the words in the train set. Comparing with original InferSent algorithm, the structure of the model is modified for PPI prediction. Conneau used GloVe (Pennington et al., 2014) word vector in InferSent, but we use GO term vectors to train Word2vec for InferSent_PN model because GO term vectors imply more semantic biological information. Conneau tried different encoder models for construction of InferSent, such as LSTM, GRU, BiLSTM with mean/max pooling, self-attentive network, and hierarchical ConvNet. Among them, BiLSTM has the best performance. In this work, InferSent_PN model utilizes the convolutional neural network (CNN) as the sentence coder since the order of words has little effect on the results of the model, and the performance of CNN is better than BiLSTM. InferSent classifies data into three classes with labels of ‘Entailment’, ‘Contradiction’, or ‘Neutral’. However, InferSent_PN classifies data into two classes, ‘positive’ and ‘negative’.

**FIGURE 4:**
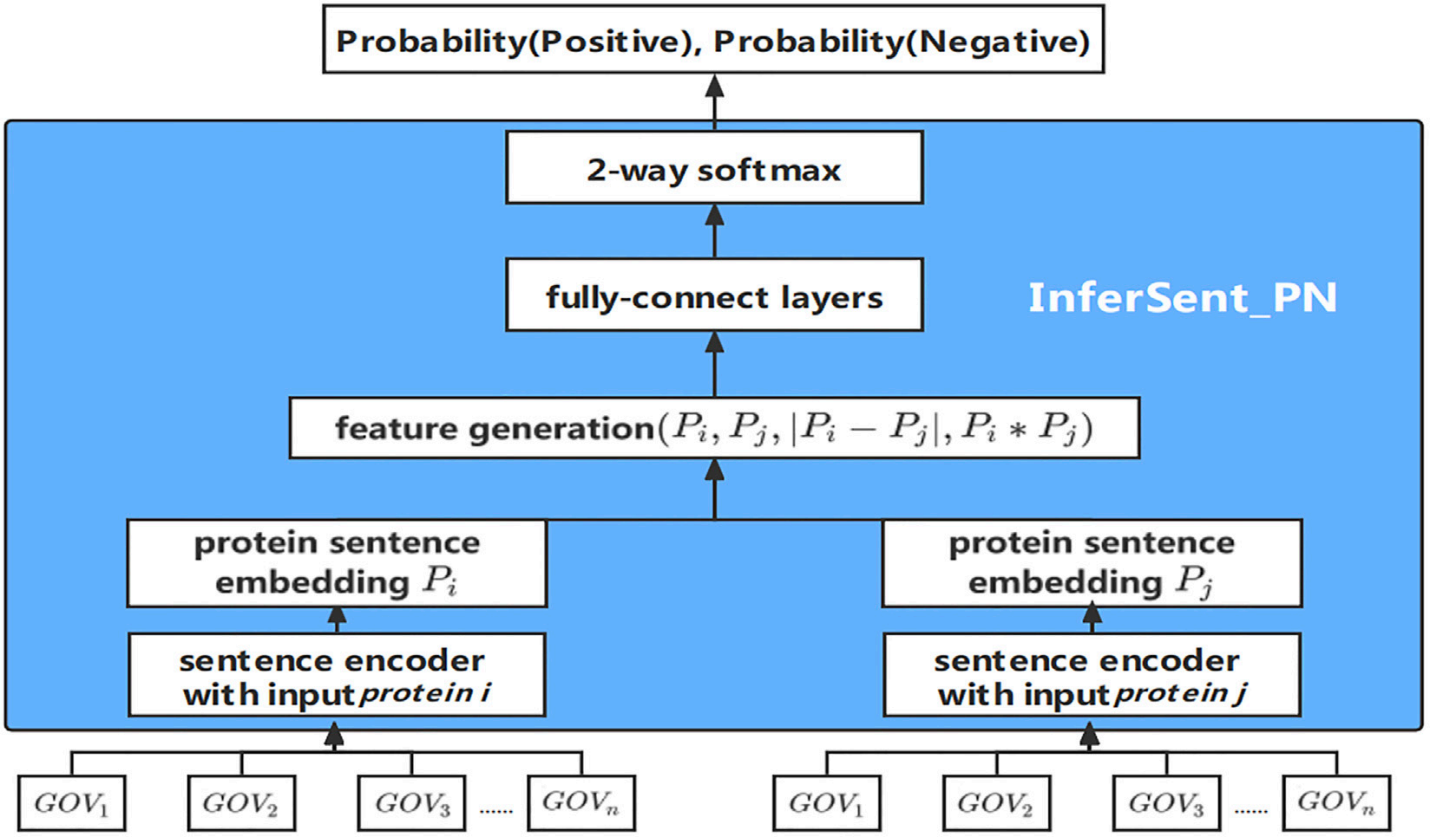
The workflow of the InferSent_PN model.

InferSent_PN method regards protein as sentence, protein sentence P, and vector representations of GO terms as word vector (GO term vectors GOV). Training data of InferSent_PN is composed of pairs of protein sentences and relationship label between protein pairs. The workflow of InferSent_PN model is shown in Figure 4. First, a pair of the sets of GOVs annotating proteins input InferSent_PN model, sets of GOVs are encoded by the sentence encoder to obtain protein sentence embedding P_i_ and P_j_. P_i_ and P_j_ embedding goes through the middle layer of extracting the features of these two vectors, and finally outputs the probability of belonging to every category in the output layer for PPI prediction.

The formula for predicting PPI is as following Eq. 1:

$$InferSent\_PN\;\left(P_{i}\;,P_{j}\right)=\;Probability\left(positive\right)>Probability\left(negative\right)\;?positive:negative$$ (1)

The input of InferSent_PN is (a set of GOVs, a set of GOVs) as (protein i, protein j). In Section 2.2.1, we introduced two versions of a method to generate annotation axioms of InferSentPPI. Based on the two methods, we have implemented two versions of the InferSentPPI method. Using “PGAA_noweight” in the InferSentPPI method is named as the “InferSentPPI_ noweight_ PGAA” method, and using “PGAA_Weight” in the InferSentPPI method is named as “InferSentPPI _ weight_ PGAA".

## Results and Discussion

### Datasets

To test the efficiency of a proposed method, seven benchmark datasets were applied in the experiments. The seven benchmark datasets are a yeast (S. cerevisiae) dataset and a human dataset from the STRING database (Damian et al., 2017), a yeast (the *S. cerevisiae* core) dataset, an *E. coli* dataset, a *Homo sapiens* dataset, and a mice dataset (Hashemifar et al., 2018) from a database of interacting proteins (DIP), and a human dataset from the human protein references databases (HPRD).

The STRING *S. cerevisiae* dataset contains 6,392 proteins and 2,007,135 interactions, and the DIP*S*. *cerevisiae* core contains 5,594 positive protein pairs and 5,594 negative protein pairs. The STRING human dataset contains 19,577 proteins and 11,353,057 interactions, and the HPRD human dataset is made up of 3,899 positive protein pairs and 4,262 negative protein pairs. Interaction pairs with reliable GO annotation records were left through the preprocessing step. Then, the dataset used for the experiment is shown inTable 1.

**TABLE 1:** The number of PPIs in seven test datasets after the preprocessing.

Database	STRING		DIP	HPRD	DIP		
Label	^#^Yeast	^#^Human	^#^Yeast	^#^Human	^#^E.coli	^#^H.sapiens	^#^M.musculus
Positive	414,240	435,209	5,436	536	1,112	981	100
Negative	414,240	435,209	5,436	536	—	—	—
Total	828,480	870,418	10,872	1,072	1,112	981	100

### Evaluation Metrics

To evaluate the performance of PPI prediction, we used six measures, including Accuracy, Precision, Recall, F1, Area Under the ROC curve (AUC_ ROC), and area under PR curve (AUC_ PR). Accuracy is the ratio of the number of samples correctly classified by the classifier to the total number of samples. Precision calculates the proportion of the number of positive samples for correct prediction to the number of samples whose prediction is positive. Recall calculates the proportion of the number of samples whose prediction is positive and correct to the number of samples that are actually positive. ROC curve and PR curve are widely used to evaluate the performance of classification and prediction tasks (A and B, 2018). ROC curve is defined by the relationship between true positive rate (TPR) and false positive rate (FPR). PR curve is defined by the relationship between Precision and Recall. Recall is the abscissa and Precision is the ordinate.

### Model Construction and Parameter Setting

We randomly selected 90% of yeast dataset and human dataset to train the InferSentPPI model. The selection of batch size has some influence on the training of the InferSentPPI model. By setting batch size = 2 in model training, InferSentPPI has the best performance on the yeast test set. In addition, by setting batch size = 1 in model training, InferSentPPI has the best performance on the human test set. So, we set the batch size to one for the yeast dataset and set the batch size to two for the human dataset.

The similarity between GO terms is calculated by three exiting methods, Resnik (Resnik, 1995), Lin (Lin, 1998), and Pekar (Pekar and Staab, 2002). The semantic similarity between two proteins are calculated based on the similarities between related GO terms by three methods, average value (AVG) (Xu et al., 2008), maximum value (Max) (Pesquita et al., 2009), and best match average (BMA) (Li et al., 2010). Compared with AVG and Max, BMA achieved the best performance. Thus, we select BMA to calculate the similarity between proteins.

According to the similarities between two terms in GO, the semantic similarity between two proteins is calculated by AVG (Xu et al., 2008), Max (Pesquita et al., 2009), and best match average (BMA) (Li et al., 2010), which are defined by Eqs 2–4:

$$Fun_{AVG}\left(p_{1},p_{2}\right)=\frac{1}{\left|T_{1}\right|\left|T_{2}\right|}\sum IC\left(\left\{t_{1},t_{2}\right\}\right)\left(t_{1}\in T_{1},t_{2}\in T_{2}\right)$$ (2)

$$Fun_{MAX}\left(p_{1},p_{2}\right)=MAX\left\{IC\left(\left\{t_{1},t_{2}\right\}\right)\right\}\left(t_{1}\in T_{1},t_{2}\in T_{2}\right)$$ (3)

$$Fun_{BMA}\left(p_{1},p_{2}\right)=\frac{1}{2}\left(\frac{1}{\left|T_{1}\right|}\sum IC\left(\left\{t_{1},t_{2}\right\}\right)+\frac{1}{\left|T_{2}\right|}\sum IC\left(\left\{t_{1},t_{2}\right\}\right)\right)\left(t\in T_{1},t_{2}\in T_{2}\right)$$ (4)

where ${\text{p}}_{1}$ and ${\text{p}}_{2}$ are the pair of proteins, ${\text{T}}_{1}$ and ${\text{T}}_{2}$ are the set of GO terms that annotate the protein ${\text{p}}_{1}$ and ${\text{p}}_{2}$ , respectively. The information content (IC) is a similarity measurement method between two terms in ontology, and the detailed calculation formula is shown in the Supplementary Material file.

### Comparison With Existing GO Structure-Based Methods

To evaluate the effectiveness of proposed methods, we compare InferSentPPI with representative GO structure-based PPI prediction methods (Resnik, 1995; Lin, 1998; Pekar and Staab, 2002; Wang et al., 2007). In the experiment, we used DIP yeast dataset and HPRD human dataset to evaluate the performance InferSentPPI method. We randomly selected 10% of the data as the test set, which is independent of train data.

Tables 2 and 3 show the evaluation results of our proposed models and the compared models on two different datasets, HPRDhuman dataset and DIPyeast dataset. The best results on each dataset are highlighted in bold. The six evaluation indicators performance of InferSentPPI on DIPyeast dataset and HPRDhuman dataset are better than four other traditional GO structure-based models, including Resnik, Lin, Wang, and Pekar. The PPI prediction method uses supervised sentence embedding technology to regard protein as sentence and vector representation of GO term as a word vector. So, it can effectively capture the relationship between proteins from a GO structure and a GO annotation for reliable PPI prediction.

**TABLE 2:** Performance comparison of six methods on the yeast dataset from DIP.

Method	Accuracy	Precision	Recall	F1	AUC_ROC	AUC_PR
Resnik_BMA	0.6957	**0.9953**	0.3933	0.5638	0.8275	0.8779
Lin_BMA	0.7794	0.7911	0.7591	0.7747	0.8435	0.8434
Wang_BMA	0.7775	0.9265	0.6029	0.7304	0.8406	0.8815
Pekar_BMA	0.7739	0.9209	0.5993	0.7260	0.8449	0.8828
InferSentPPI_noweight_PGAA	0.9476	0.9371	0.9595	0.9481	0.9868	0.9884
InferSentPPI_weight_PGAA	**0.9522**	0.9346	**0.9724**	**0.9531**	**0.9915**	**0.9921**

**TABLE 3:** Performance comparison of six methods on the human dataset from HPRD.

Method	Accuracy	Precision	Recall	F1	AUC_ROC	AUC_PR
Resnik_BMA	0.611	**1**	0.222	0.3633	0.7661	0.815,999
Lin_BMA	0.7129	0.6666	0.8518	0.7479	0.7918	0.785,539
Wang_BMA	0.6851	0.7941	0.5	0.6136	0.7475	0.799,019
Pekar_BMA	0.75	0.8859	0.574	0.6966	0.8024	0.791,993
InferSentPPI_noweight_PGAA	0.8796	0.8727	0.8888	0.8806	0.9540	0.9544
InferSentPPI_weight_PGAA	**0.9444**	0.9166	**0.9565**	**0.9361**	**0.9686**	**0.9679**

On the DIP yeast and HPRD human datasets, five leading evaluation indicators of InferSentPPI_weight_GOA are better than InferSentPPI_unique_GOA. It means the model’s performance generated on a corpus with weighted GO annotations is better than the model generated on a corpus with weightless GO annotations. The result indicates that the quantitative index of GO annotation reliability successfully provides valuable information for PPI prediction.

**FIGURE 5:**
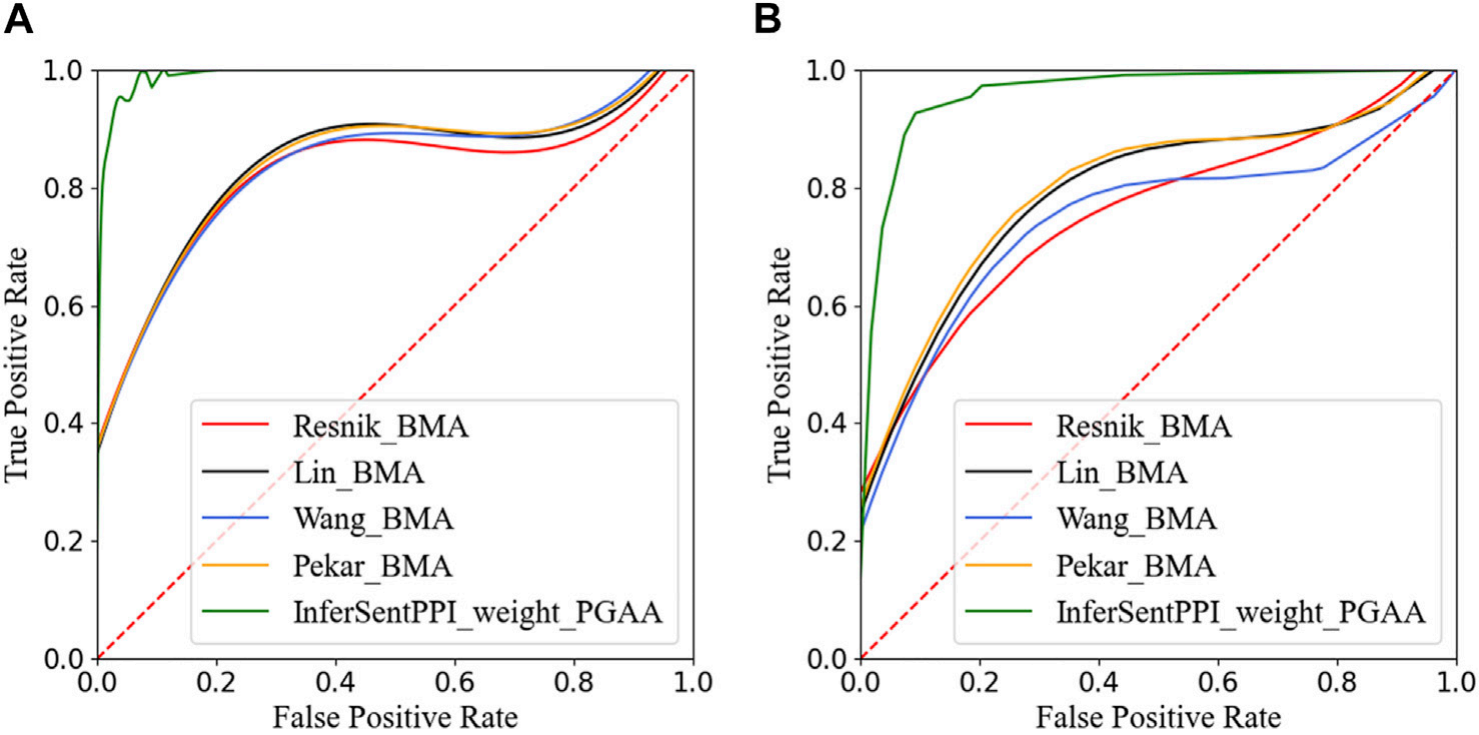
ROC curves of five PPI prediction methods on the main dataset. **(A)** Yeast dataset from DIP. **(B)** Human dataset from HPRD.

Figure 5 reports the ROC curves of our model and four traditional GO structure-based models on DIP yeast dataset and HPRD human dataset. The AUC_ROC of the two methods on DIP yeast and HPRD human data sets reached 0.99 and 0.96. From the results, we noticed that the InferSentPPI method is stable in predicting both positive and negative datasets. AUC_ ROC is usually applied to evaluate the model’s classification performance, which is independent of the selected threshold. The results show that the proposed method still effectively classifies the datasets under different thresholds.

### Comparison With State-Of-The-Art GO Information-Based Methods

To evaluate the effectiveness of proposed methods, we compare InferSentPPI with state-of-the-art GO information-based PPI prediction methods, Onto2Vec (Smaili et al., 2018) and GO2Vec (Zhong et al., 2019). In this experiment, we used yeast and human datasets from STRING to test the performance of InferSentPPI and other existing methods.

The performance comparison results of the methods are shown in Table 4. The best result on each dataset is highlighted in bold. The AUC_ROC of the two methods on yeast and human datasets from STRING reached 0.8745 and 0.8233. The result shows that the performance of InferSentPPI is better than two state-of-the-art GO information-based PPI prediction methods.

**TABLE 4:** AUC_ROC of three GO Information-based methods on yeast and human dataset from STRING.

Method	AUC_ROC	
	STRING Yeast	STRING Human
Onto2Vec	0.7660	0.7593
GO2Vec_mhd_goa	0.8154	0.8046
InferSentPPI_weight_PGAA	**0.8745**	**0.8233**

### Comparison With a State-Of-The-Art Sequence-Based Method

To deeply evaluate the effectiveness of proposed methods, we compare InferSentPPI with a state-of-the-art sequence-based method DeepFE-PPI (Yao et al., 2019). The result is shown in Table 5. In the experiment, we used the DIP yeast dataset and HPRD human dataset to evaluate the performance InferSentPPI method. We also randomly selected 10% of the data as the test set independent of train data.

**TABLE 5:** Performance comparison of three methods on the DIP yeast and HPRD human datasets.

Data	Method	Accuracy	Precision	Recall	F1	AUC_ROC	AUC_PR
Human	DeepFE_PPI	**0.9871**	**0.9877**	**0.9854**	**0.9865**	—	—
	InferSentPPI_noweight_PGAA	0.8796	0.8727	0.8888	0.8806	0.9540	0.9544
	InferSentPPI_weight_GOA	0.9444	0.9166	0.9565	0.9361	**0.9686**	**0.9679**
Yeast	DeepFE_PPI	0.944	**0.9652**	0.9212	0.9426	0.9821	0.9854
	InferSentPPI_noweight_PGAA	0.9476	0.9371	0.9595	0.9481	0.9868	0.9884
	InferSentPPI_weight_GOA	**0.9522**	0.9346	**0.9724**	**0.9531**	**0.9915**	**0.9921**


The number of PPIs in the DIP yeast dataset used in the experiment is 10 times larger than the HPRD human dataset. On the DIP yeast dataset, the four evaluating indicators of the two methods of the InferSentPPI are better than the sequence-based PPI prediction method DeepFE-PPI. However, neither of the two methods of the InferSentPPI outperforms DeepFE-PPI on the HPRD human dataset, which is much smaller than the DIP yeast dataset. The experiment result shows that the InferSentPPI performs better than DeepFE_PPI when there is sufficient training data.

### Performance Comparison on Independent Species-specific PPI Datasets

To sufficiently evaluate the generalization and robustness of the InferSentPPI model, the model from the first experiment, trained on the DIP yeast dataset, is used to predict PPI on three species-specific PPI datasets (*E. coli, H. sapiens*, mice) (Zhou et al., 2011).

On three species-specific PPI datasets, Table 6 reports the accuracy of the InferSentPPI model, which is trained on the yeast dataset from the first experiment. In Table 6, the model’s accuracy is 0.9522 on the yeast test set, and the performance of this model on the PPI test set of other species is also stable. In addition, the accuracy of this model on the mouse dataset reaches 0.95, including 100 positive records, which is smaller than the others. On the *E. coli* positive dataset, including 1,112 records, the accuracy of our model also reaches 0.90. The availability of our model in predicting multiple species is proved. It means that the InferSentPPI method can obtain a better generalization model from a single species data set with sufficient data.

**TABLE 6:** Performance (accuracy) of InferSentPPI on different independent datasets.

Dataset	Accuracy
Yeast	0.9522
*M. musculus*	0.95
*H. sapiens*	0.8974
*E. coli*	0.9073

## Conclusion

Accurate prediction of PPI can help us understand the underlying molecular mechanisms and significantly promote drug discovery. The method based on GO information can be used to make reliable PPI predictions. In this paper, we apply the modified supervised sentence embedding model InferSent to mine GO information and PPI data, used to predict PPIs. We used seven different datasets to evaluate our method to thoroughly test the InferSentPPI model. Compared with representative GO information-based methods and a sequence-based PPI prediction method, the experimental results show the effectiveness and generalization of the InferSentPPI method. The result also indicates that the quantitative index of GO annotation reliability successfully provides valuable information for PPI prediction. “PGAA_ Weight” can improve the performance of PPI prediction.

## Data Availability

The original contributions presented in the study are included in the article/Supplementary Material, further inquiries can be directed to the corresponding author.
