# Supplementary material for: InfersentPPI: Prediction of Protein-Protein Interaction Using Protein Sentence Embedding With Gene Ontology Information
Source: Front Genet. 2022 Mar 28;13:827540. doi: 10.3389/fgene.2022.827540 (PMC8995897; doi:10.3389/fgene.2022.827540)
Supplement: Supplementary file 1 [file DataSheet1.docx]

Supplementary Material

## A Functional similarity between GO terms

# Node-based method

Resnik calculates semantic similarity based on the information content (IC) of the terms in ontology. The IC of *t* term is defined by  Equation 1 (Lee et al., 2004):

$\text{IC}(t)=-\log p(t)$ (1)

Here $p\left( t \right)$ is the probability of the occurrence of the *t* term.

Resnik (Resnik, 1995) calculated the similarity as Equation 2:

$\text{Sim}_{\text{Resnik }}\left(t_{1},t_{2}\right)=-\log p\left(t_{m}\right)$ (2)

Among them, $t_{m}$ is the common ancestor of the most abundant IC values of $t_{1}$ and $t_{2}$ in ontology.

Lin (Lin, 1998) calculated the similarity as Equation 3:

$\text{Sim}_{\text{Lin}}\left(t_{1},t_{2}\right)=\frac{2*\log p\left(t_{m}\right)}{\log p\left(t_{1}\right)+\log p\left(t_{2}\right)}$ (3)

# Edge-based method

Pekar (Pekar and Staab, 2002) calculated the similarity as  Equation 4:

$\text{Sim}_{P\text{ekar}}\left(t_{1},t_{2}\right)=\frac{\delta\left(t_{a},\text{ }root\text{ }\right)}{\delta\left(t_{a},\text{ }root\text{ }\right)+\delta\left(t_{1},\text{ }root\text{ }\right)+\delta\left(t_{2},\text{ }root\text{ }\right)}$ (4)

Where $\delta\left( t_{1},t_{2} \right)$ denotes the longest distance between term $t_{1}$ and $t_{2}$ in GO graph, and $t_{a}$ is Least Common Ancestor (LCA) of $t_{1}$ , $t_{2}$. Three distances are used in the equation.

# Hybrid method

Wang (Wang et al., 2007) calculated the similarity as  Equation 5, 6 and 7:

$\left\{\begin{matrix} S_{A}(t)=1 & \text{ }if\text{ }t=A \\ S_{A}(t)=MAX\left\{w_{e}*SA\left(t^{'}\right)\text{∣}t^{'}\in\text{ }childrenof(t)\right\} & \text{ }if\text{ }t\neq A \end{matrix}\right.$ (5)

$SV(A)=\sum S_{A}(t)(t\in T_{A})$ (6)

$S_{GO}(A,B)=\frac{\sum\left(S_{A}(t)+S_{B}(t)\right)}{SV(A)+SV(B)}(t\in T_{A}\cap T_{B})$ (7)

Factor and the fixed weight assigned by the edge. $S_{A}\left( t \right)$ represents the contribution value of any term *t* to the semantics of term *A*. $T_{A}$ is the set of all ancestors of GO term *a*, while $E_{A}$ is the set of corresponding links. $w_{e}$ represents the term contribution factor of edge *e* connecting term *T* and its sub term *t* in $E_{A}$. $w_{e}$ represents the term contribution factor of edge *e* connecting term *t* and *t's* sub term *t'* in $E_{A}$. $SV\left( A \right)$ represents the semantic value of GO term *A*, and $S_{GO}\left( A,B \right)$ is the semantic similarity measure of GO term *A* and *B*.

# References

Lee, S.G., Hur, J.U., and Kim, Y.S. (2004). A graph-theoretic modeling on GO space for biological interpretation of gene clusters. *Bioinformatics* 20(3)**,** 381-388.

Lin, D. (1998). "An information-theoretic definition of similarity", in: *Proceedings of the 15th International Conference on Machine Learning*, 296-304.

Pekar, V., and Staab, S. (2002). "Taxonomy learning-factoring the structure of a taxonomy into a semantic classification decision", in: *COLING 2002: The 19th International Conference on Computational Linguistics*).

Resnik, P. (1995). "Using information content to evaluate semantic similarity in a taxonomy," *In: Proceedings of the International Joint Conference for Artificial Intelligence,* 448–453.

Wang, J.Z., Du, Z., Payattakool, R., Yu, P.S., and Chen, C.-F. (2007). A new method to measure the semantic similarity of GO terms. *Bioinformatics* 23(10)**,** 1274-1281.
